# Supplementary material for: Nanoscale imaging of super-high-frequency microelectromechanical resonators with femtometer sensitivity
Source: Nat Commun. 2023 Mar 2;14:1188. doi: 10.1038/s41467-023-36936-9 (PMC9981767; doi:10.1038/s41467-023-36936-9)
Supplement: Supplementary file 1 — Supplementary Information [file 41467_2023_36936_MOESM1_ESM.pdf]

Supplementary Information

Nanoscale Imaging of Super-High-Frequency Microelectromechanical Resonators with Femtometer Sensitivity

Daehun Lee[1], Shahin Jahanbani[1], Jack Kramer[2], Ruochen Lu*[2], Keji Lai*[1]

[1] Department of Physics, University of Texas at Austin, Austin TX 78712, USA
[2] Department of Electrical and Computer Engineering, University of Texas at Austin, Austin TX 78712, USA

* E-mails: ruochen@utexas.edu, kejilai@physics.utexas.edu

Supplementary Note 1. Characterization of the LOBAR device.

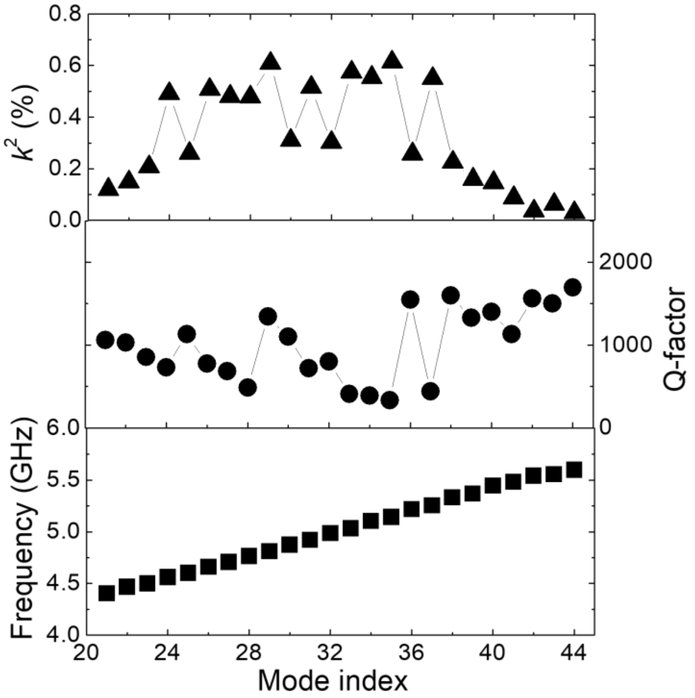

Supplementary Figure. 1 | Key parameters of the LOBAR device. Extracted parameters of the resonator, including the electrotechnical coupling, Q-factors, and resonant frequencies as a function of the mode index.

Fig. S1 shows the key parameters of the LOBAR device extracted by using the recursive multi-resonance MBVD model [Ref. S1] and fitting to the measured admittance curve (Fig. 1b in the main text). Fluctuations of the parameters are presumably due to the slight mode distortion caused by the IDTs and device imperfections.

Supplementary Note 2. Spatial resolution of TMIM

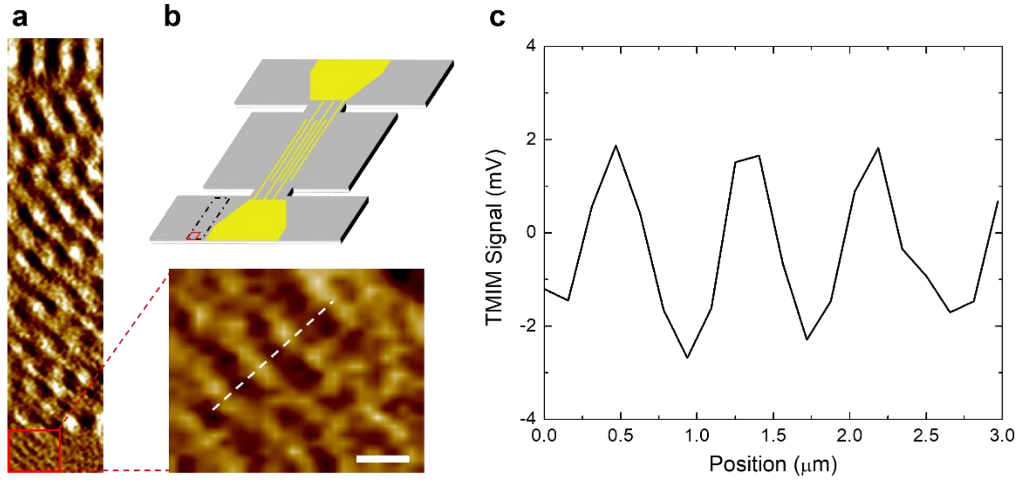

Supplementary Figure. 2 | Small features resolved by the TMIM. a TMIM image near the anchored area. The bottom region is unsuspended and clamped by the Si substrate (see Fig. 1a in the main text). **b** Schematic of the LOBAR device with the region in (**a**) marked by the dash-dotted rectangle. The inset in the bottom is a close-up view of the red box in (**a**). The scale bar is 1 μm. **c** TMIM signal across the dashed line in (**b**). The acoustic wave with a wavelength of ~ 0.8 μm is clearly resolved.

Fig. S2a shows the TMIM image near an electrode of the LOBAR device. The bottom region of the $LiNbO_3$ thin film is unsuspended and clamped by the Si substrate. The phase velocity drops from ~ 12 km/s on the freestanding film to ~ 4 km/s on the unreleased film, resulting in a wavelength of ~ 0.8 μm here. The close-up image in Fig. S2b and the line profile in Fig. S2c both indicate that the TMIM can easily resolve lateral features on the order of 100 nm.

Supplementary Note 3. Complete set of TMIM data at various overtones

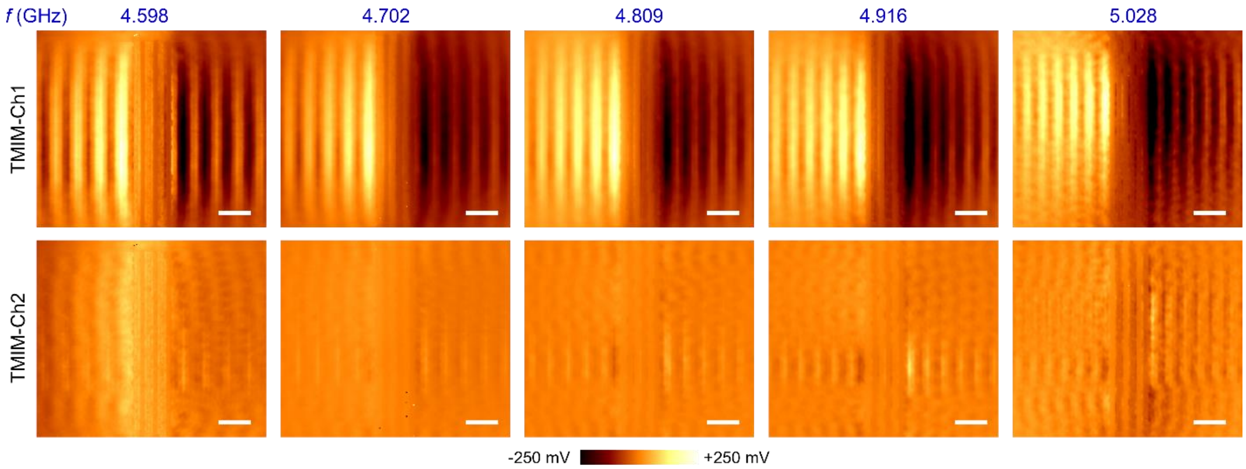

Supplementary Figure. 3 | Complete set of TMIM data at 5 overtones. Left to right: TMIM-Ch1 and -Ch2 images taken at the 25th, 27th, 29th, 31st, and 33rd overtones. All scale bars are 5 μm.

The complete TMIM images at different overtones are shown in Fig. S3. For standing waves in this LOBAR device, we can put most signals to a single TMIM channel, in this case TMIM-Ch1, by adjusting the mixer phase [Ref. S2]. As a result, we only present data in this channel for Figs. 3-5 in the main text.

Supplementary Note 4. Impedance match of TMIM tip for sensitivity measurement

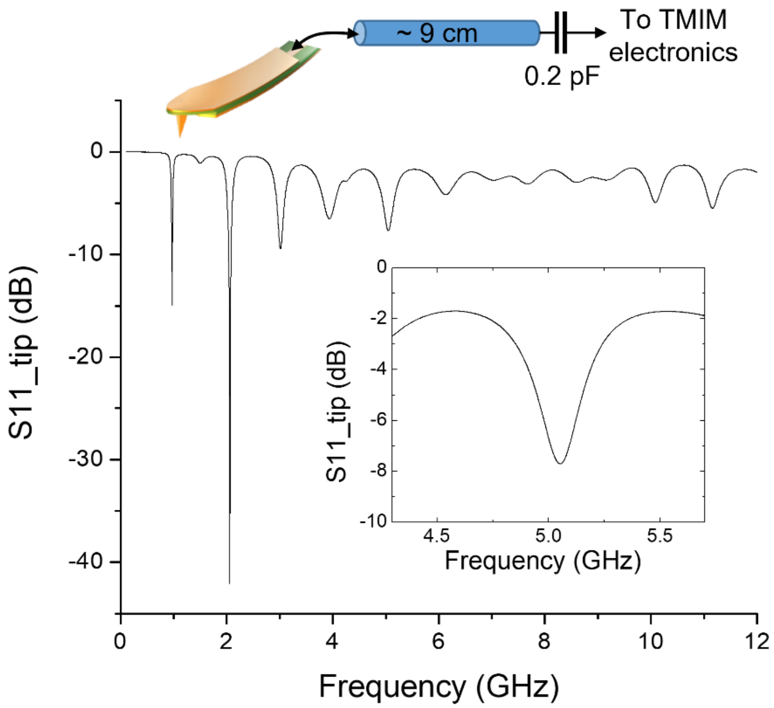

Supplementary Figure. 4| Impedance-match for the TMIM tip. S11 measured by VNA after impedance (*Z*) match of the TMIM tip, with zoom-in data near 5 GHz shown in the inset. The schematic of the *Z*-match network is shown on the top.

In order to cover a broad frequency range of the LOBAR device, we directly connected the TMIM tip to electronics in most experiments in this work. For the evaluation of the ultimate sensitivity (Fig. 5 in the main text), however, we matched the impedance of the TMIM tip to 50 Ω transmission lines (inset of Fig. S4) to further enhance the detection sensitivity [Ref. S2]. The S11 of TMIM tip after the impedance match is shown in Fig. S4. The sensitivity floor near 5 GHz is improved by a factor of ~ 3 due to the *Z*-match.

Supplementary Note 5. TMIM data at low input power and comparison with FEA simulation

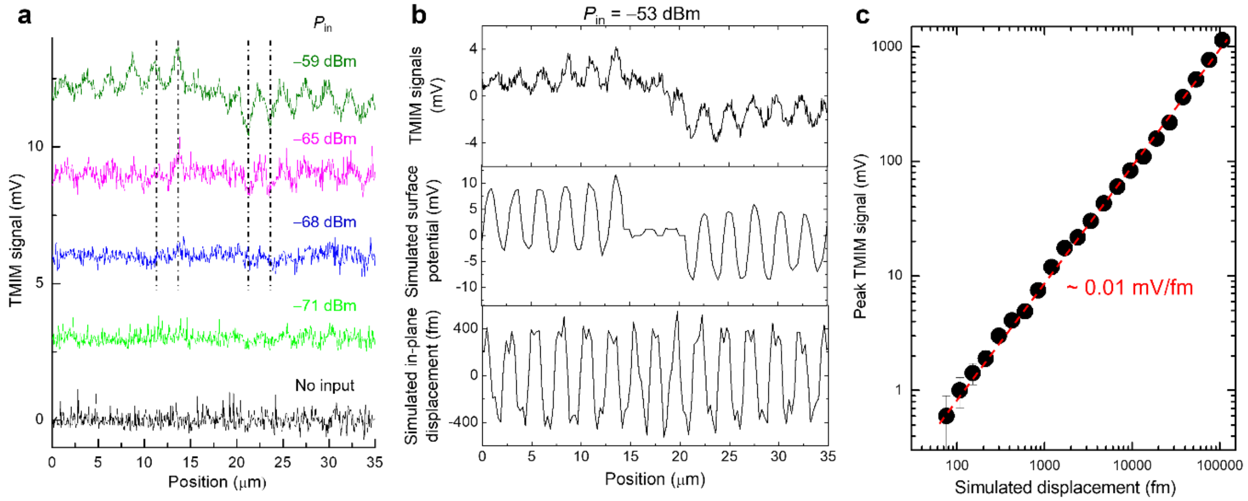

Supplementary Figure. 5 | Comparison between TMIM signals and FEA simulation. a TMIM line profiles at small or no input power. The dash-dotted lines mark the peak positions near the electrodes. **b** Top to bottom: Measured TMIM signals, simulated piezoelectric potential, and simulated in-plane displacement at a higher P_{in} = –53 dBm. **c** Peak TMIM signals versus simulated oscillation amplitude at all input powers. The red dashed line is a linear fit to the data with a slope ~ 0.01 mV/fm.

Fig. S5a shows the TMIM signals at low input powers near the noise floor. As marked by the dash-dotted lines, the peaks near electrodes are well resolved at P_{in} = –65 dBm, just resolved at –68 dBm, and barely discernible at –71 dBm, which are consistent with Fig. 5b in the main text. Note that since the acoustic signal at P_{in} = –68 dBm is just above the noise level, a direct comparison between simulation and experiment here is not compelling. In Fig. S5b, we plot the TMIM data, simulated piezoelectric potential, and simulated in-plane displacement at a higher P_{in} = –53 dBm, where the acoustic signals are much more prominent. The resemblance between the FEA results and TMIM data supports the claimed match between simulation and experiment. Finally, by comparing the peak TMIM signals and the simulated amplitude of in-plane displacements at all input powers, we obtain the conversion factor of ~ 0.01 mV/fm in our current setup, as seen in Fig. S5c.

References:

S1. Lu, R., Li, M.-H., Yang, Y., Manzaneque, T. and Gong, S. Accurate Extraction of Large Electromechanical Coupling in Piezoelectric MEMS Resonators. *J. Microelectromechanical Syst.* **28**, 209 (2019).

S2. Zheng, L., Wu, D., Wu, X. and Lai, K. Visualization of surface-acoustic-wave potential by transmission-mode microwave impedance microscopy. *Phys. Rev. Appl*. **9**, 061002 (2018).
